# Anti-Inflammatory Profile of *Jungia sellowii* Less. by Downregulation of Proinflammatory Mediators and Inhibition of NF-*κ*B and p38 Pathways

**DOI:** 10.1155/2020/9078956

**Published:** 2020-04-11

**Authors:** Geison Vicente, Yeo Jim Kinoshita Moon, Daniela Weingärtner Rosa, Luíse Azevedo Lima, Najla Adel Saleh, Julia Salvan da Rosa, Tânia Beatriz Creczynski-Pasa, Maique Weber Biavatti, Eduardo Monguilhott Dalmarco, Tânia Silvia Fröde

**Affiliations:** ^1^Department of Clinical Analysis, Center of Health Sciences, Federal University of Santa Catarina, Florianópolis, Santa Catarina, Brazil; ^2^Department of Pharmaceutical Sciences, Center of Health Sciences, Federal University of Santa Catarina, Florianópolis, Santa Catarina, Brazil

## Abstract

*Jungia sellowii* Less. (Asteraceae) is a native plant found in Southeast Brazil used traditionally to treat inflammatory diseases. This study was conducted (1) to investigate the toxicity of the crude extract (CE) and (2) to investigate the mechanism of the anti-inflammatory action of *J. sellowii* L. roots. The potential acute toxicity of CE was performed by administration of only different doses of CE (500, 1,000, and 2,000 i.p.) on mice for 14 days. The anti-inflammatory effect was evaluated using carrageenan-induced acute pleural cavity inflammation in a mouse model, evaluated through the following inflammatory variables: leukocyte, protein concentrations of the exudate, myeloperoxidase (MPO), adenosine deaminase (ADA), nitric oxide metabolites (NO_x_), and proinflammatory cytokine (tumor necrosis factor alpha (TNF-*α*), interferon gamma (IFN-*γ*), interleukin- (IL-) 6, and IL-12) levels in mouse pleural fluid leakage. The p65 protein phosphorylation of nuclear factor NF-kappa B (p65 NF-*κ*B) and p38 mitogen-activated protein kinase (p38 MAPK) phosphorylation were analyzed in lung tissue. Our results demonstrated that the administration of CE up to 2,000 mg/kg did not present a toxic effect. In addition, the pretreatment of mice with CE; its derived fractions (aqueous fraction (AqF), butanol fraction (BuOHF), and ethyl acetate fraction (EtOAcF)); and isolated compounds (curcuhydroquinone *O*-*β*-glucose (CUR) and *α* and *β* piptizol (Pip)) reduced the following inflammatory variables: neutrophils, protein concentrations of the exudate, MPO, ADA, NO_x_, and proinflammatory cytokine (TNF-*α*, IFN-*γ*, IL-6, and IL-12) levels in mouse pleural fluid leakage. The compounds CUR and Pip also decreased the p65 protein phosphorylation of NF-kappa B and p38 (MAPK) in lung tissue. *J. sellowii* L. has important anti-inflammatory activity with potential applications in drug development against inflammatory disorders. These effects found can be attributed to the ability of the new isolated compounds CUR and Pip to suppress p65 NF-*κ*B and p-p38 MAPK pathways.

## 1. Introduction

The control of mediators released in the inflammatory process ensures the anti-inflammatory effectiveness. The limitations of current therapies with uncontrolled or unresolved inflammation result in tissue damage with eventual loss of organ function, characteristic of chronic inflammatory diseases [1, 2].

The mouse model of pleurisy induced by carrageenan (Cg) is a well-characterized inflammation model which allows the quantification of different proinflammatory mediators released into the pleural fluid leakage of the pleural cavity [3]. This model is characterized by infiltration of neutrophils 4 h after pleurisy induction, followed by lung injury by reactive oxygen species (ROS) and reactive nitrogen species (RNS) released from active neutrophils such as hydrogen peroxide (H2O2), superoxide anion (O2−), hydroxyl radical (OH−), and peroxynitrite (ONOO−) [4].

Although a broad spectrum of anti-inflammatory drugs is currently available, a new strategy for the identification of new targets and discovery of new molecules, particularly to treat diseases resistant to conventional treatment with an inflammatory nature, must be done [5, 6].

Natural products remain an important source for drug discovery and structural diversity of new substances or prototypes with relevant therapeutic potential, and many anti-inflammatory drugs have their origin in extracts or compounds from plants [6].

*Jungia* (Asteraceae) is a perennial herb that comprises shrubs, lianas, and herbs recorded from Mexico to the Andes and also present in Brazilian dry forests [7]. In traditional Brazilian folk medicine, in Andean communities at the middle north of Perú, and in traditional medicine of Ecuador, species of the genus *Jungia* (*J. paniculata* and *J. rugosa*) are used in tea form (decoction) to treat inflammation of the liver and kidneys and of the digestive system, to treat urinary tract inflammations, and also as an aseptic agent for treating wounds [8, 9].

Moreover, pharmacological studies have reported the anti-inflammatory and antioxidant properties of various species of *Jungia*. Recently, analysis of Wilches et al. [10] demonstrated the potential anti-inflammatory of methanolic extract from *J. rugosa* leaves, using an acute model of croton oil ear edema and carrageenan-induced paw edema in rats. Also, the anti-inflammatory activity was demonstrated in a chronic inflammation model using cotton pellet-induced granuloma in mice.

Casado et al. [11] demonstrated the antioxidant and anti-inflammatory activities of polyphenols and flavonoids from *Jungia paniculata* using *in vitro* (phospholipase A2 (sPLA_2_) inhibition assay) and *in vivo* (carrageenan-induced paw edema in rats and 12-O-tetradecanoylphorbol-13-acetate- (TPA-) induced ear edema in mice) models.

However, there are few past studies concerning the anti-inflammatory effect of *Jungia sellowii* Less. Thus, the present study is aimed at investigating the toxicity of *J. sellowii* L. roots using *in vivo* and *in vitro* assays and at analyzing the anti-inflammatory effect of *J. sellowii* L. roots using the classical *in vivo* experimental model of acute inflammation. In this protocol, we evaluated the mechanism of the anti-inflammatory action of the crude extract (CE), its derived fractions, and isolated compounds by evaluating several proinflammatory variables such as leukocyte influx, protein concentrations of the exudate, myeloperoxidase and adenosine deaminase, nitric oxide metabolite (NO_x_) levels, and Th1 polarization immune response through analysis of the level of proinflammatory cytokines: tumor necrosis factor alpha (TNF-*α*), interferon gamma (IFN-*γ*), interleukin- (IL-) 6, and IL-12 cytokines. Furthermore, the effect of the newly isolated compounds (curcuhydroquinone *O*-*β*-glucose (CUR) and *α* and *β* piptizol (Pip)) upon p65 protein phosphorylation of nuclear factor-kappa B (p65 NF-*κ*B) and p38 of mitogen-activated protein kinase MAPK (p38 MAPK) phosphorylated levels in the lungs was also investigated.

## 2. Materials and Methods

### 2.1. Plant Material

The roots of *Jungia sellowii* L. were collected from the Rio Negrinho county, Santa Catarina, Brazil, during March, 2012. The plant material was authenticated by Dr. Ademir Reis of the Botanic Department at the Federal University of Santa Catarina. A voucher specimen (RB 537991) was deposited in the Herbarium Dimitri Sucre Benjamin-Botanical Garden in Rio de Janeiro (Botanical Garden), Brazil.

### 2.2. Obtainment of Crude Extract and Fractions

The roots of *J. sellowii* L. (3.0 kg) were macerated with ethanol (EtOH) 70° Gl for 3 d in the absence of light. This procedure was repeated three times at room temperature (approximately 25°C) for obtainment of the crude extract (CE). Subsequently, the CE was dissolved in H_2_O (1/3) and subjected to liquid–liquid partitioning with the solvents hexane, dichloromethane, ethyl acetate, and butanol. The resulting fractions were concentrated to dryness in a rotary evaporator to obtain hexane (HexF) (3 g), dichloromethane (DCMF) (1.2 g), ethyl acetate (EtOAcF) (2.9 g), butanol (BUOHF) (16 g), and aqueous (AqF) (94 g) fractions. The isolated compounds (CUR and Pip) were obtained from the ethyl acetate (2.9 g) and butanol (16 g) fractions, respectively.

### 2.3. Qualitative Profile Analysis of Ethyl Acetate and Butanol Fractions by UPLC

Ultraperformance liquid chromatography (UPLC) analysis was performed using an Acquity H-Class UPLC system (Waters Co.) equipped with a photodiode array detector (PDA). The chromatographic separation was achieved using an Acquity UPLC BEH C18 column (50 × 2.1 mm^2^, 1.7 *μ*m) at a flow rate of 250 *μ*L/min with an injection volume of 2 *μ*L. The mobile phase consisted of a gradient system combining 0.1% aqueous formic acid (pH 3.0) (A) and acetonitrile (B). The phase for the ethyl acetate fraction comprised 0–5 min 65% (A), 5–6 min 98% (B), and 6–7 min 65% (A), whereas for the butanol fraction, the phase used was 0–5 min 80% (A), 5–6 min 90% (B), 6–6.1 min 98% (B), and 6.1–7 min 80% (A).

### 2.4. Structural Elucidation

The isolated compounds were subjected to spectrometric analysis by nuclear magnetic resonance (NMR) hydrogen (^1^H) and carbon (^13^C). The equipment used included (1) the Varian AS400, model 400 MHz to 100 MHz for ^1^H and ^13^C (source: Middelburg, Holland) of the Chemistry Department at Federal University of Santa Catarina, Brazil; (2) the BRUKER brand, model DRX 400 Avance, ^1^H frequency of 400 MHz and ^13^C of 100.62 MHz (Source: Bruker Daltonik, Bremen, Germany) of the Chemistry Department of the Federal University of Paraná, Brazil; and (3) Bruker AC-300 and Bruker Avance-400 at 400 MHz for 2D ^1^H experiments and 75 MHz for ^13^C (Source: Bruker Daltonik, Bremen, Germany) of the Pharmacognosy Department at Université René Descartes, France.

### 2.5. In Vitro Study

#### 2.5.1. Cell Culture

Murine macrophage J774 cell line was maintained in 75 cm^2^ bottles in 5% carbon dioxide (CO_2_) atmosphere at 37°C in Dulbecco's Modified Eagle's Medium (DMEM), pH 7.4, supplemented with 10% fetal bovine serum (FBS). Cells were passaged in subconfluence by standard trypsinization procedure, and viable cells are checked in the beginning of experiment by trypan blue exclusion test.

#### 2.5.2. Cytotoxicity

The cell viability was assessed using 3-(4,5-dimethiazol-zyl)-2-5-diphenyltetrazolium bromide (MTT) assay in a J774 cell density of 1.0 × 10^4^ cells/well, as previously described by Mosmann [12].

Briefly, the plate with 200 *μ*L of cell suspension was incubated at 37°C under a CO_2_ condition for 24 h to cell adhesion. Posteriorly, cells were incubated with the respective control saline solution (NaCl, 0.9%), control dimethyl sulfoxide (DMSO) vehicle-treated, or CE of *J. sellowii* L. at concentrations of 1 mg/mL, 0.1 mg/mL, 0.01 mg/mL, and 0.001 mg/mL. At the end of the experiment (24 h), the supernatant was removed, and fresh medium was added containing 10% (*v*/*v*) of MTT solution (5 mg/mL) in phosphate-buffered saline (PBS) (pH 7.6, composition: NaCl, 130 mmol; Na_2_HPO_4_, 5 mmol; and KH_2_PO_4_, 1 mmol). The plate was incubated for 2 h at 37°C, and the supernatant was removed posteriorly. Subsequently, 100 *μ*L of a solution of pure DMSO was added to each well and the plate was agitated for 10 min. The supernatant was read at 540 nm in an enzyme-linked immunosorbent assay (EIA) plate reader (Organon Teknika, Roseland, NJ, USA). The optical density of the control DMSO vehicle-treated was regarded as 100% of viable cells. The cytotoxic concentration to 50% of cells (CC_50_) was determined using a nonlinear regression analysis (log of concentration versus response) by Prism 5.0 GraphPad software.

### 2.6. In Vivo Study

#### 2.6.1. Animals

Female Swiss mice weighing 20–22 g were used for all experiments. The animals were kept in a room at a constant temperature of 22 ± 2°C with alternating 12 h periods of light and darkness at 50–60% humidity. The mice were kept in plastic cages with water and food provided *ad libitum* before the experiments. The present study was approved by the Committee for Ethics in Animal Research at the Federal University of Santa Catarina, Brazil (License No. 4720140616). All animal experiments were conducted in accordance with the International guidelines (NIH publication #85-23, revised in 1985), as well as the guidelines of the Brazilian College of Animal Experimentation (CONCEA).

#### 2.6.2. Pleurisy Induced by Carrageenan

Carrageenan-induced pleurisy in mice is a useful model, for testing natural products with potential anti-inflammatory activity and for further understanding their mechanism of action.

Pleurisy was induced by a single intrapleural (i.pl.) injection of 0.1 mL Carrageenan (Cg, 1%), as previously described by Saleh et al. [3]. After 4 h, the animals were killed with an overdose of ketamine and xylazine administered by intraperitoneal route (i.p.), the thorax was opened, and the pleural cavity was washed with 1.0 mL of sterile phosphate-buffered saline (PBS, pH 7.6, composition: NaCl, 130 mmol; Na_2_HPO_4_, 5 mmol; and KH_2_PO_4_, 1 mmol) in distilled water containing heparin (20 IU/mL). Samples of the mice pleural fluid leakage were collected to analyze leukocyte influx, protein concentration of the exudate, myeloperoxidase (MPO), adenosine deaminase (ADA), metabolites of nitric oxide (NO_x_), and cytokine (TNF-*α*, IFN-*γ*, IL-6, and IL-12) levels in mouse pleural fluid leakage. Lung tissue samples were collected for determination of the total/phosphorylated levels of p65 NF-*κ*B and p38 MAPK.

#### 2.6.3. Acute Toxicity

The acute toxicity test for the hydroalcoholic extract of *J. sellowii* L. roots was conducted to evaluate any possible toxicity. In this protocol, four groups of five female mice were divided into a control group and test groups. The negative group received an injection of sterile saline solution (NaCl, 0.9% i.p.) (vehicle), and the second to fourth group received different doses of CE of *J. sellowii* L. (500, 1,000, or 2,000 mg/kg, i.p.) This was considered the start of the test (day 0). The general behavior and condition of the animals (tremors, convulsions, diarrhea, sleep, coma, mortality, and weight lost) were observed and registered from 0.25 to 3 h after the herb injection and daily for 14 d. On day 14, the animals were sacrificed and vital organs such as the kidney, spleen, liver, and lungs were isolated and weighed (humidity weight).

Hematology variables including white blood cells (WBC), lymphocytes, mononuclear cells, granulocytes, erythrocytes (RBC), hemoglobin, hematocrit, mean corpuscular volume (MCV), mean corpuscular hemoglobin (MCH), mean corpuscular hemoglobin concentration (MCHC), red cell distribution width (RDW), and platelet count were also analyzed on day 14 using a veterinarian automatic counter (Mindray, BC-2800 Vet, Nanshan, Shenzhen, China).

#### 2.6.4. Pleurisy Experimental Design

For the analysis of the dose-response curve, the animals were randomly divided into 11 groups of five animals: group 1 (negative control) animals that received only an intrapleural (i.pl.) injection of sterile saline (NaCl, 0.9%); group 2 (positive control) animals that received only Cg (1%, i.pl.); group 3 animals that received an intraperitoneal (i.p.) injection of dexamethasone (Dex: 0.5 mg/kg) plus Cg; and groups 4 to 11 comprised animals which received i.p. injections of different doses of CE (25–200 mg/kg) or its derived fractions (AqF (10–50 mg/kg), BuOHF (5–25 mg/kg), DCMF (10–50 mg/kg), HexF (10–50 mg/kg), and EtOAcF (5–25 mg/kg)) or the isolated compounds (CUR (1–5 mg/kg) and Pip (0.5–5 mg/kg)), administered 0.5 h prior to pleurisy induction with Cg. The inflammatory variables neutrophil and mononuclear leukocytes and protein concentrations of the exudate were analyzed 4 h after Cg administration.

To determine the time course profile, different groups of animals were pretreated at different time points (0.5 to 2 h) with only one dose of CE (lower doses that inhibited leukocytes and/or protein concentrations of the exudate). Inflammation was analyzed 4 h after Cg administration. The optimal time of pretreatment was the lower time of pretreatment required to inhibit leukocytes and/or protein concentrations of the exudate. This period of pretreatment applied to the CE was extended to the fractions and isolated compounds.

Thus, based on the results of these experiments the following doses were chosen: 50 mg/kg for CE, 25 mg/kg for AqF, 10 mg/kg for BuOHF, 10 mg/kg for EtOAcF, 2.5 mg/kg for CUR, and 1 mg/kg for Pip, all administered 0.5 h before Cg induction. These doses were selected to analyze their effects upon other proinflammatory variables such as MPO, ADA, and NO_x_ and the levels of cytokines (TNF-*α*, IFN-*γ*, IL-6, and IL-12) in the mouse pleural fluid leakage. The phosphorylation of p65 NF-*κ*B and p38 MAPK levels was analyzed in the mouse lung tissues.

Dexamethasone (0.5 mg/kg, i.p.) administered 0.5 h before pleurisy induction was used as the reference anti-inflammatory drug.

#### 2.6.5. Quantification of Cell Migration and Protein Concentrations of the Exudate

The total leukocyte counts from mouse pleural fluid leakage were performed using a veterinarian automatic counter (Mindray, BC-2800 Vet, Nanshan, Shenzhen, China), and the cytospin preparations were stained with May-Grünwald-Giemsa for the differential leukocyte count. The protein concentrations of the exudate were indirectly determined by the quantity of Evans blue dye extravasation in the mouse pleural fluid leakage [3]. Thus, in each experimental group, the animals were challenged 0.5 h before the Cg injection with a solution of Evans blue dye (25 mg/kg) administered intravenously (i.v.) to evaluate the protein concentrations of the exudate. The amount of dye in the pleural wash aliquot was estimated via colorimetric measurements using an enzyme immune assay (EIA) plate reader (Organon Teknika, Roseland, NJ, USA) at 620 nm by interpolation from a standard curve of Evans blue dye (in the range of 0.01–50 *μ*g/mL).

#### 2.6.6. Quantification of Myeloperoxidase (MPO) Levels

In-house assays for the MPO were employed according to the methodology described in the literature by Rao et al. [13]. The colorimetric analysis was performed using an EIA plate reader (Organon Teknika, Roseland, NJ, USA) at 450 nm and interpolated from a standard curve of MPO from human neutrophils (Sigma: M6908, St. Louis, MO, USA) ranging from 0.7 to 140 mU/mL. The results were expressed in mU/mL.

#### 2.6.7. Quantification of Adenosine-Deaminase (ADA) Levels

A 20 *μ*L sample from the mouse pleural fluid leakage was used to determine the ADA levels using a previously described methodology by Giusti and Galanti [14]. This analysis was performed calorimetrically using an EIA plate reader (Organon Teknika, Roseland, NJ, USA) at 630 nm and estimated by an ammonium sulfate solution, measured in triplicate as a standard point of 20 U/L on a colorimetric plate reader (Organon Teknika). The results were expressed in U/L.

#### 2.6.8. Quantification of Nitric Oxide Metabolite (NO_X_) Levels

Quantification of the nitric oxide product (nitrite (NO_2_^−^) and nitrate (NO_3_^−^)) levels was determined according to the methodology of Griess [15]. Levels of nitrite were estimated by interpolation from a standard curve of sodium nitrite (0–150 *μ*M) by colorimetric measurements at 540 nm in an EIA plate reader (Organon Teknika, Roseland, NJ, USA). The results were expressed as *μ*M.

#### 2.6.9. Quantification of the Levels of TNF-*α*, IFN-*γ*, IL-6, and IL-12

The determination of the TNF-*α*, IFN-*γ*, IL-6, and IL-12 levels was performed with aliquots of the washed mouse pleural fluid leakage using a commercial kit for flow cytometric CBA (cytometric bead array) mouse inflammation (BD Biosciences Pharmingen, San Diego, CA, USA), according to manufacturer's instructions. The measurements were performed in a FACSVerse flow cytometer (BD FACSVerse™, San Jose, CA, USA), and the data were analyzed in FCAP Array software version 3.0, from the same manufacturer. The limits of detection were 7.3 pg/mL, 2.5 pg/mL, 5 pg/mL, and 10.7 pg/mL for TNF-*α*, IFN-*γ*, IL-6, and IL-12, respectively. The intra-assay precision was 3–4%, 3–5%, 4–5%, and 4–15% for TNF-*α*, IFN-*γ*, IL-6, and IL-12, respectively. The interassay precision was 6–11%, 5–8%, 8–11%, and 3–9% for TNF-*α*, IFN-*γ*, IL-6, and IL-12, respectively. The values were expressed in pg/mL.

#### 2.6.10. Quantification of Total p65 Protein and Phosphorylated (p-p65 NF-*κ*B) Levels

The evaluation of p65 protein (NF-*κ*B) was performed in the lung tissue. Samples were collected and the total protein concentration was adjusted to 60 *μ*g using the method described by Lowry and Lewis (1951). In this protocol, commercial kits were used with monoclonal antibodies specific for total and phosphorylated p65 (PathScan®-NF-*κ*B p65 (Ser536)) EIA Kit (Cell Signaling Technology, Inc., Danvers, Massachusetts, USA) following the instructions of the manufacturer. The absorbance of the samples was measured calorimetrically using an EIA plate reader (Organon Teknika, Roseland, NJ, USA) at 450 nm. The results were expressed as the relative fold change in comparison with the negative control group, which represented the basal level of p65 phosphorylation.

#### 2.6.11. Evaluation of the Total and Phosphorylated p38 protein (P-p38 MAPK)

The evaluation of the p38 protein (p38 MAPK) was performed in the lung tissue. The total protein concentrations of the lung samples were adjusted to 60 *μ*g according to method developed by Lowry and Lewis (1951). In this experimental protocol, commercial kits with monoclonal antibodies specific for total and phosphorylated p38 were used (Instant One® Phospho-p38 MAPK (Tyr180/Tyr182) and eBioscience®, San Diego, CA, USA) using EIA assays following the instructions of the manufacturer. From the obtained absorbance, a relative analysis comparing the results with the negative control group was performed, which represents the baseline concentrations of phosphorylated p38.

### 2.7. Statistical Analysis

To assess data normality and the homogeneity of variances, the Shapiro–Wilk test was used. The normal distributions of variables were summarized as means ± standard error of the mean (SEM), and categorical variables were expressed as percentage of five animals per group. For the statistical analyses of the results (animals treated with phlogistic agent vs. animals treated with *J. sellowii* L.), a test of analysis of variance (ANOVA) was used followed by a Newman-Keuls post hoc test. For all analyses, *p* < 0.05 was considered significant, and all data were analyzed by the statistical program GraphPad version 5.0 (San Diego, CA, USA).

## 3. Results

### 3.1. Ultraperformance Liquid Chromatography (UPLC-PDA) of *J. sellowii* L. Fractions

**Figure 1:**
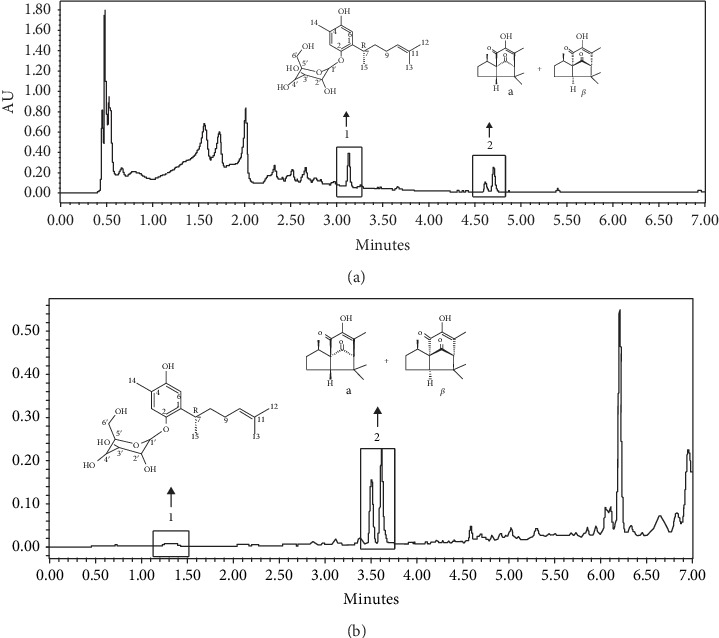
(a) Fingerprint (UPLC) of the butanol fraction from *J. sellowii* L. at 280 nm, number 1: curcuhidroquinone *O*-*β*-glucose. (b) Fingerprint (UPLC) of the ethyl acetate fraction from *J. sellowii* L. at 285 nm, number 2: *α* and *β* piptizol.

**Figure 2:**
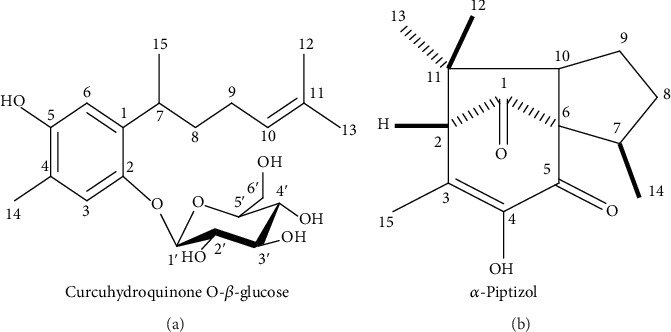
(a) The chemical structure of the isolated compounds: compound 1—curcuhidroquinone *O*-*β*-glucose isolated from butanol fraction of *J. sellowii* L roots; (b) compound 2—piptizol isolated from the ethyl acetate fraction of *J. sellowii* L. roots.

The UPLC method was employed at 280 nm and 285 nm for the identification of the compound majority in the fractions. The chromatogram of the butanol fraction showed peak 1 identified as curcuhydroquinone *O*-*β*-glucose (MW 396 g/mol), chemical name 2-(4-hydroxy-5-methyl-2-(6-methylhept-5-en-2-yl) phenoxy)-6-(hydroxymethyl) tetrahydro-2H-pyran-3,4,5-triol, with a retention time of 3.13 min, and the second peak was identified as beta-piptizol and alpha-piptizol (MW 248 g/mol), chemical name (3R, 3aR)-5-hydroxy-3,6,8,8-tetramethyl-2,3,8,8a-tetrahydro-1H-3a, 7-methanoazulene-4,9 (7H)-dione, with a retention time of 4.52-4.62 min (Figure 1(a)). The ethyl acetate fraction is shown in the first peak CUR with a retention time of 1.38 min and the second peak the Pip with a retention time of 3.50-3.61 (Figure 1(b)). The identified pure substances CUR and Pip in the butanol and ethyl acetate fractions were performed by comparison of retention time with known standards (Figures 2(a) and 2(b)).

### 3.2. Cytotoxicity In Vitro Test

**Figure 3:**
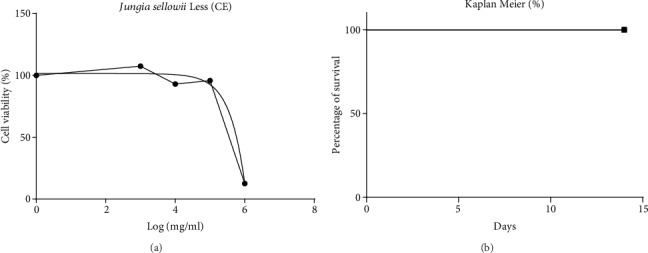
(a) Percentage of cell viability of CE of *J. sellowii* L. upon macrophages J774 in the test of MTT (3-(4,5-dimethiazol-zyl)-2-5-diphenyltetrazolium bromide); (b) survival curve of animals treated with CE of *J. sellowii* L. The values in the graphs of experiments *in vitro* represent the mean of three experiments, and the results of experiments *in vivo* are presented as percentage of survival of 5 mice compared to the control group saline (NaCl, 0.9%) for 14 days of observation.

Initially, a concentration-response evaluation of the cytotoxic effects of *J. sellowii* L. was performed. Murine macrophage J774 cell line was incubated with indicated concentrations of CE (1, 0.1, 0.01, and 0.001 mg/mL) for 24 h. Results of the MTT-based assay of control DMSO vehicle-treated cells were defined as 100% of viable cells, and cells incubated with *J. sellowii* L. were presented as a relative decrease on viable cells with increasing concentration (Figure 3(a)). Based on the results, the cytotoxic concentration was calculated, which is the concentration able to kill 50% of cells (CC_50_). For the CE of *J. sellowii* L., the CC_50_ was 580 *μ*g/mL.

### 3.3. Acute Toxicity Test

The CE of *J. sellowii* L. (i.p.) administered in doses of 500 mg/kg, 1,000 mg/kg, or 2,000 mg/kg did not cause any mortality up to 2,000 mg/kg (Figure 3(b)). Signs and symptoms which occurred in response to a high dose of the extract were a decrease in motor activity and weight loss with diarrhea. After two days of CE administration, the mice that received the high dose (2,000 mg/kg) returned to normal without demonstrating any signs and symptoms of toxicity. The internal organs (kidney, spleen, liver, and lungs) of both the control (NaCl, 0.9%) and CE-treated groups did not show any unusual signs, and the organs were shown to be normal in size, weight, and color (*p* > 0.05) (results not shown). The hematological variables also did not differ from the control group and in animals pretreated with CE of *J. sellowii* L. after 14 d (*p* > 0.05) (results not shown).

### 3.4. Effects of Extract, Fractions, and Isolated Compounds of *J. sellowii* L. upon Leukocyte Influx and Protein Concentration of the Exudate

The CE (50 to 200 mg/kg) significantly reduced leukocytes (*p* < 0.01), neutrophils (*p* < 0.01), and protein concentrations of the exudate (*p* < 0.01). However, the same effect was not observed upon mononuclear cells (*p* > 0.05) (Table 1).

The AqF (25 to 50 mg/kg) also showed an important anti-inflammatory effect by inhibiting leukocytes (*p* < 0.01) and neutrophils (*p* < 0.01). Furthermore, this fraction at doses of 10 to 50 mg/kg showed significant inhibition of protein concentrations of the exudate (*p* < 0.01). However, no alteration of mononuclear cells was observed (*p* > 0.05) (Table 1).

A similar anti-inflammatory effect was observed for BuOHF and EtOAcF. Both fractions at doses of 10 to 25 mg/kg inhibited leukocytes (*p* < 0.01), neutrophils (*p* < 0.01), and protein concentrations of the exudate (*p* < 0.01). Again, no change was observed in relation to mononuclear cells (*p* > 0.05) (Table 1).

HexF only inhibited the protein concentrations of the exudate at a dose of 25 mg/kg (*p* < 0.05), and the dichloromethane fraction (DCMF) did not inhibit the studied inflammatory variables (*p* > 0.05). Therefore, the effect of these fractions upon other inflammatory variables was not studied (results not shown).

In relation to the isolated compounds CUR (2.5–2.5 mg/kg) and Pip (1–5 mg/kg), both significantly inhibited leukocytes (*p* < 0.01) and neutrophils (*p* < 0.01). Furthermore, CUR (1–5 mg/kg) and Pip (0.5–5 mg/kg) inhibited protein concentrations of the exudate (*p* < 0.05). CUR (2.5 mg/kg) increased mononuclear cells (*p* < 0.01), whereas Pip showed no change to these cells (*p* > 0.05) (Table 1).

The reference anti-inflammatory drug Dex significantly inhibited the tested inflammatory variables (*p* < 0.01) (Table 1).

### 3.5. Effects of Extract, Fractions, and Isolated Compounds of *J. sellowii* L. upon Myeloperoxidase and Adenosine-Deaminase Levels

The pretreatment (0.5 h) of animals with selected doses of CE (50 mg/kg); its derived fractions AqF (25 mg/kg), BuOHF (10 mg/kg), and EtOAcF (10 mg/kg); and the isolated compounds CUR (2.5 mg/kg) and Pip (1 mg/kg) significantly decreased both the MPO and ADA levels (*p* < 0.01) (Table 2). Dex also reduced this proinflammatory variable (*p* < 0.01) (Table 2).

### 3.6. Effects of Extract, Fractions, and Compounds of *J. sellowii* L. upon NO_X_ Concentrations

Because *J. sellowii* L. caused a significant decrease of the protein concentrations of the exudate, we investigated whether this inhibition could be associated with a decrease in NO_x_ levels. The results showed that CE; its derived fractions AqF (25 mg/kg), BuOHF (10 mg/kg), and EtOAcF (10 mg/kg); and the isolated compounds CUR (2.5 mg/kg) and Pip (1 mg/kg) significantly decreased NO_x_ levels (*p* < 0.01) (Table 2). Dex also reduced this proinflammatory variable (*p* < 0.01) (Table 2).

### 3.7. Effects of Extract, Fractions, and Compounds of *J. sellowii* L. on TNF-*α*, IFN-*γ*, IL-6, and IL-12 Levels

The results demonstrated that CE (50 mg/kg) and the derived fractions AqF (25 mg/kg), BuOHF (10 mg/kg), and EtOAcF (10 mg/kg) and the isolated compounds CUR (2.5 mg/kg) and Pip (1 mg/kg) of *J. sellowii* L. significantly decreased the levels of the cytokines TNF-*α* (*p* < 0.05), IFN-*γ* (*p* < 0.01), IL-6 (*p* < 0.01), and IL-12 (*p* < 0.01) (Table 3). Dex also reduced these cytokine levels (*p* < 0.01) (Table 3).

### 3.8. Effect of the Isolated Compounds on Total and Phosphorylated p65 Protein Levels (p-p65 NF-*κ*B)

**Figure 4:**
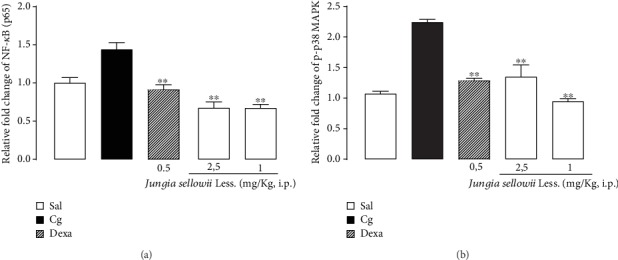
(a) Effects of the isolated compounds curcuhidroquinone *O*-*β*-glucose (CUR: 2.5 mg/kg) and piptizol (Pip: 1 mg/kg) from *J. sellowii* L. roots administered 0.5 h prior to carrageenan (1% Cg, i.pl.) on total and phosphorylated protein (p-p65 of NF-*κ*B) in the lungs; (b) effects of the isolated compounds curcuhidroquinone *O*-*β*-glucose (CUR: 2.5 mg/kg) and piptizol (Pip: 1 mg/kg) from *J. sellowii* L. roots administered 0.5 h prior to carrageenan (1% Cg, i.pl.) on total and phosphorylated protein (p-p38 MAPK) in the lungs. Sal: negative control group, animals treated only with sterile saline (NaCl, 0.9%); Cg: positive control group, animals treated only with 1% Cg; Dex: animals pretreated with dexamethasone (0.5 mg/kg). The results were expressed in relative fold change in comparison to Sal, which represents the basal level of p65 and p38 MAPK phosphorylation. Bars represent the mean ± SEM of five animals. ANOVA: Newman-Keuls test.

Therefore, we investigated if the compound isolates from *J. sellowii* L. could act on this signaling pathway. CUR (2.5 mg/kg) and Pip (1 mg/kg) significantly decreased the NF-*κ*B-p65 phosphorylation (% of inhibition: CUR, 56.80 ± 5.13%; Pip, 56.88 ± 4.07%) (*p* < 0.01), but not the total p65 NF-*κ*B levels (Figure 4(a)). The same inhibition was observed in animals pretreated with Dex (0.5 mg/kg) (*p* < 0.01) (Figure 4(b)).

### 3.9. Effect of *J. sellowii* L. on Total and Phosphorylated p38 Protein (p38 MAPK) Levels

The compounds CUR (2.5 mg/kg) and Pip (1 mg/kg) also decreased p-p38 MAPK, but not the total p38 MAPK (% of inhibition: CUR, 39.83 ± 11.33% and 57.91 ± 2.83%) (*p* < 0.01) (Figure 4(b)). Similarly, the pretreatment of the animals with the reference drug Dex reduced p-p38 MAPK (*p* < 0.01), but not the total p38 MAPK levels (Figure 4(b)).

## 4. Discussion

Several important *in vivo* and *in vitro* models have been developed with an experimental value and clinical payoffs.

Cytotoxicity *in vitro* assay showed a high CC_50_ value (580 *μ*g/mL) of CE of *J. sellowii* L. for murine macrophage J774 cell line, demonstrating a good result to this study, since the intention was not to kill macrophages but just to modulate the inflammation, as they play an important role in inflammation and in the presentation of antigens to lymphocytes [16]. Thus, the CE of *J. sellowii* L. supposedly proved promising for inflammatory *in vivo* study evaluation.

Acute toxicity studies *in vivo* were evaluated by the organ weight, by changes in behavior, and by the index of physiological status of the animal, considering increase organ, organomegaly, or inflammation as a strong indicator. Furthermore, together with hematological variables, these are considered sensitive parameters evaluated in studies with toxic plants [17, 18]. Based on the results, roots of *J. sellowii* L. extract in acute treatment by i.p. route at a dose of up to 2000 mg/kg did not produce deaths during 14 d of observation ensuring the doses in experiments to evaluate the anti-inflammatory activity.

Based on these previous studies that do not report evidence of toxicity, we evaluated the effect of CE, fractions obtained from CE (AqF, BuOHF, EtOAcF, HEX-F, and DMC-F), and isolated compounds (CUR and Pip) on the levels of several proinflammatory mediators to investigate the anti-inflammatory mechanism of action of *J. sellowii* L. using a typical mouse model of pleurisy induced by carrageenan.

Many of the classic experiments in the field of inflammation have been performed using murine [19]. Carrageenan-induced pleurisy in mice is a useful model, for testing natural products with potential anti-inflammatory activity and also for further understanding their mechanism of action [3]. The results of our study show that *J. sellowii* L. demonstrated a pronounced inhibition on leukocyte migration due in part to CE and polar fractions AqF, BuOHF, and EtOAcF and compounds CUR and Pip have the ability to reduce neutrophil migration.

The amplitude of leukocyte activation and migration can be modulated by the proinflammatory enzymes such as MPO and ADA and mediators such as NO_x_ and proinflammatory cytokine levels (TNF-*α*, IFN-*γ*, IL-6, and IL-12) important in the context perpetuation of inflammatory response.

MPO is a hemoprotein located in azurophil granules of neutrophils and has been used as a biochemical marker for neutrophil infiltration into tissues and tissue damage. MPO activity is also directly related to the amount of leucocyte infiltration, which is indicative of ROS and RNS release from activated neutrophils and the hypochlorous acid (HOCL) production, which in large quantities leads to the injury of the host cells [20].

Corroborating our results, Wilches et al. [10] observed that leaves of *J. rugose* showed an anti-inflammatory effect by reducing the MPO levels, as well as neutrophil accumulation at doses of 125 to 500 mg/kg on the croton oil-induced model in mice.

Moreover, ADA is another molecule that has emerged for the management of several inflammatory conditions in preclinical and clinical settings. The adenosine system plays a crucial part in the regulation of immune homeostasis, acting as a self-limiting signal aimed to preserve the host integrity through immune cell activation and promoting the resolution of inflammation [21].

The inhibition of the ADA enzyme promotes the increase of extracellular adenosine from activated cells such as neutrophils and macrophages. This adenosine binds to the A2A receptors in an autocrine/paracrine manner, thereby promoting the inhibition of intercellular adhesion molecule 1 (ICAM-1) and preventing cell infiltration, proinflammatory cytokine release, neutrophil degranulation, and oxidative stress in the lungs [22].

The formation of protein of the exudate is another important sign of inflammation, and the metabolites of NO play a role in the exudative phase during the inflammatory process. Interferons (IFNs), cytokines, and microbial products are prototypic transcriptional inducers of NOS_2_ which effectively stimulate the release of high amounts of nitric oxide. The released nitrite/nitrate plays an important role as a vasodilator and contributes to increase of edema, thereby contributing to the formation of (ONOO−) and promoting important tissue injury [23].

Furthermore, the proinflammatory cytokines IL-6, TNF-*α*, IFN-*γ*, and IL-12 play such an important inductor of inflammation in disorders such as severe/uncontrolled asthma, Alzheimer's disease, and rheumatoid arthritis and may be considered a predictive factor for treatment failure [24]. Thus, we investigated if *J. sellowii* L. could modulate the Th1 immune response by analysis of certain cytokines released into the inflamed mouse pleural fluid leakage. The results indicate an increase of these cytokines induced by carrageenan administration that was significantly inhibited by the CE, AqF, BuOHF, and EtOAcF of *J sellowii* L.

The effects on cytokine levels involve a complex intracellular process. NF-*κ*B is an important self-control of cytokine expression. The nuclear transcription factor NF-*κ*B plays a role as a principal mediator to physiological processes and in response to pulmonary injury, and several inflammation processes regulate the innate immunity and inflammation. NF-*κ*B controls the expression of proinflammatory cytokines and other effector proteins and enzymes involved in immune response such as inducible nitric oxide synthase (iNOS) and cyclooxygenase 2 (COX-2) [25].

The classical pathway of NF-*κ*B activation depends on the activation of the I*κ*B kinase (IKK) complex, formed by two catalytic subunits IKK*α* and IKK*β* and a regulatory unit NEMO. IKK*β* promotes the phosphorylation of I*κ*B*α*. Activation of the classical NF-*κ*B pathway depends on the p65/p50 heterodimer, in which the p65 subunit phosphorylation leads to the transmigration of the NF-*κ*B activated form to the nucleus, where the gene expression of proinflammatory mediators is triggered [26].

In addition, p38 MAPK is considered an important regulator of multiple responses during the inflammatory process. The superfamily of highly conserved kinases regulates cell growth, differentiation, and stress responses. During inflammation, p38 MAPK regulates distinctly different functions, including the chemotaxis of neutrophils through chemoattractants (CXCL8, CXCL2, and LTB4), molecules of endothelial and leukocyte-associated adhesion, activation of NF-*κ*B, synthesis and release of ROS, and cytokines such TNF-*α* when they are induced by carrageenan [27].

Thus, based on our results, we showed for the first time that the compounds isolated from the polar fractions, CUR and Pip, were effective in reducing the influx of neutrophils and production of proinflammatory mediators *in vivo*, at least in part, due to the ability to reduce the phosphorylation of p65 NF-*κ*B subunit and p38 MAPK.

The compound curcuhydroquinone *O*-*β*-glucose is a new molecule described as being similar to bisabolane sesquiterpene isolated from the rhizome of the plant *Curcuma xanthorrhiza*. This molecule shows anti-inflammatory and antioxidant proprieties preventing erythema, edema, and skin changes mediated by ultraviolet light B (UVB) by reduction of metalloproteinase-1 (MMP-1) messenger RNA (mRNA) expression in human keratinocytes [28, 29].

Piptizol is other molecule from the ethyl acetate fraction of *J. sellowii* L. roots, described such as an epimeric mixture of two isomers, *α* and *β* piptizol. There are relatively few approaches to *α* and *β* piptizol, but this mixture is considered the product of thermal rearrangement of perezone. For the *Nassauvieae* tribe, the first isolation report was from the roots of *Perezia cuernavacana*; however, there is currently no biological approach related to activity data for this compound in the literature [30, 31].

## 5. Conclusions

In summary, this study suggests that *J. sellowii* L. has an important anti-inflammatory propriety by inhibiting leukocyte influx, protein concentration of the exudate, and the liberation of several proinflammatory mediators. The results showed that *J. sellowii* L. was effective in inhibiting acute inflammation by downregulation of NF-*κ*B and p-p38 MAPK activation pathways by the isolated compounds (Pip and CUR). In addition, CE did not show a significant toxic activity, supporting its use in Brazilian folk medicine for treating inflammatory disorders.

## Figures and Tables

**Table 1:** Effects of the crude extract (CE) of *Jungia sellowii* Less., its derived fractions (aqueous (AqF), butanol (BuOHF), ethyl acetate (EtOAcF)), and isolated compounds (curcuhidroquinone *O*-*β*-glucose (CUR) and Piptizol (Pip)), upon leukocyte, neutrophil, mononuclear, and protein concentrations of the exudate, in the inflammation induced by carrageenan in the mouse model of pleurisy.

Groups	Doses (mg/kg)	Leukocytes (×10^6^) (% of inhibition)	Neutrophils (×10^6^) (% of inhibition)	Mononuclear (×10^6^) (% of inhibition)	Protein concentrations (*μ*g/mL) (% of inhibition)
Sal	0.9%^a^	1.56 ± 0.11	0.22 ± 0.03	1.41 ± 0.11	2.94 ± 0.39

Cg	1%^a^	5.46 ± 0.10	4.57 ± 0.10	0.87 ± 0.05	11.19 ± 0.87

CE	25^b^	5.24 ± 0.16	4.53 ± 0.16	0.71 ± 0.03	9.32 ± 0.94
	50^b^	3.82 ± 0.18 (30.02 ± 3.30%)^∗∗^	3.15 ± 0.16 (31.03 ± 3.51%)^∗∗^	0.67 ± 0.08	8.08 ± 0.55 (27.76 ± 4.95%)^∗∗^
	100^b^	2.60 ± 0.11 (52.37 ± 2.11%)^∗∗^	1.67 ± 0.04 (63.46 ± 0.90%)^∗∗^	1.02 ± 0.05	6.17 ± 0.54 (44.91 ± 4.85%)^∗∗^
	200^b^	2.40 ± 0.07 (56.04 ± 1.30%)^∗∗^	2.03 ± 0.16 (55.54 ± 3.57%)^∗∗^	0.53 ± 0.07	6.49 ± 0.52 (42.02 ± 4.68%)^∗∗^

AqF	10^b^	5.18 ± 0.24	4.25 ± 0.23	0.92 ± 0.06	6.59 ± 0.35 (41.13 ± 3.17%)^∗∗^
	25^b^	3.59 ± 0.11 (34.24 ± 1.93%)^∗∗^	2.82 ± 0.11 (38.38 ± 2.44%)^∗∗^	0.77 ± 0.01	5.40 ± 0.34 (51.76 ± 3.04%)^∗∗^
	50^b^	3.16 ± 0.28 (42.11 ± 5.17%)^∗∗^	2.17 ± 0.20 (5.95 ± 4.34%)^∗∗^	0.96 ± 0.09	6.21 ± 0.83 (44.47 ± 7.46%)^∗∗^

BuOHF	5^b^	5.12 ± 0.09	4.26 ± 0.10	0.85 ± 0.06	11.50 ± 0.65
	10^b^	4.15 ± 0.26 (23.98 ± 4.81%)^∗∗^	3.58 ± 0.21 (21.58 ± 4.59%)^∗∗^	0.78 ± 0.13	7.61 ± 0.65 (31.98 ± 5.79%)^∗∗^
	25^b^	3.48 ± 0.23 (36.18 ± 4.18%)^∗∗^	3.11 ± 0.22 (31.95 ± 4.88%)^∗∗^	0.67 ± 0.07	7.46 ± 0.73 (33.30 ± 6.50%)^∗∗^

EtOAcF	5^b^	5.11 ± 0.32	4.36 ± 0.33	0.75 ± 0.01	9.60 ± 1.35
	10^b^	3.33 ± 0.25 (38.96 ± 4.53%)^∗∗^	2.63 ± 0.22 (42.41 ± 4.86%)^∗∗^	0.70 ± 0.03	8.13 ± 0.97 (34.34 ± 6.63%)^∗∗^
	25^b^	3.19 ± 0.29 (41.56 ± 5.39%)^∗∗^	2.53 ± 0.28 (44.55 ± 6.13%)^∗∗^	0.64 ± 0.04	6.72 ± 0.58 (39.91 ± 5.17%)^∗∗^

CUR	1^b^	5.40 ± 0.42	4.46 ± 0.39	0.94 ± 0.06	6.94 ± 0.33 (38.03 ± 2.97%)^∗∗^
	2.5^b^	4.02 ± 0.09 (26.42 ± 1.67%)^∗∗^	2.73 ± 0.12 (40.23 ± 2.65%)^∗∗^	1.28 ± 0.12 (47.51 ± 13.74%)^∗∗^	3.91 ± 0.49 (65.06 ± 4.42%)^∗∗^
	5^b^	4.10 ± 0.24 (24.49 ± 4.41%)^∗∗^	2.95 ± 0.15 (35.45 ± 3.42%)^∗∗^	1.14 ± 0.09	4.81 ± 0.36 (57.02 ± 3.36%)^∗∗^

Pip	0.5^b^	5.10 ± 0.13	4.23 ± 0.14	0.87 ± 0.04	9.34 ± 0.30 (16.53 ± 2.67%) ^∗^
	1^b^	3.5 ± 0.25 (35.89 ± 4.60%)^∗∗^	2.73 ± 0.25 (40.31 ± 5.45%)^∗∗^	0.77 ± 0.05	6.71 ± 0.57 (40.0 ± 5.08%)^∗∗^
	2.5^b^	3.26 ± 0.16 (40.28 ± 2.87%)^∗∗^	2.30 ± 0.22 (49.67 ± 4.77%)^∗∗^	0.96 ± 0.08	4.99 ± 0.25 (55.42 ± 2.21%)^∗∗^
	5^b^	2.78 ± 0.25 (49.01 ± 4.50%)^∗∗^	2.19 ± 0.20 (51.93 ± 4.78%)^∗∗^	0.59 ± 0.04	5.95 ± 0.43 (46.89 ± 3.87%)^∗∗^

Dex	0.5^b^	2.79 ± 0.18 (48.80 ± 4.31%)^∗∗^	2.24 ± 0.10 (50.93 ± 2.82%)^∗∗^	0.55 ± 0.08	6.10 ± 0.17 (45.49 ± 1.93%)^∗∗^

Crude extract (CE: 25-200 mg/kg) of *Jungia sellowii* Less., aqueous fraction (AqF 10-50 mg/kg), butanol fraction (BuOHF 5-25 mg/kg), ethyl acetate fraction (EtOAcF: 5-25 mg/kg), curcuhidroquinone *O*-*β*-glucose (CUR: 1-5 mg/kg), and piptizol (Pip: 0.5-5 mg/kg) administered 0.5 h before pleurisy induction by carrageenan (1%). Sal: animals treated only with sterile saline solution (NaCl, 0.9%); Cg: animals treated only with carrageenan (1%); Dex: animals pretreated with dexamethasone (0.5 mg/kg); ^a^administered by intrapleural injection (i.pl.); ^b^administered by intraperitoneal route (i.p.). Each group represents the mean ± SEM of 5 animals compared to the positive control group (Cg); ANOVA/Newman-Keuls's test. ^∗^*p* < 0.05; ^∗∗^*p* < 0.01.

**Table 2:** Effects of the crude extract (CE) of *Jungia sellowii* Less., its derived fractions (aqueous (AqF), butanol (BuOHF), ethyl acetate (EtOAcF)), and isolated compounds (curcuhidroquinone *O*-*β*-glucose (CUR) and piptizol (Pip), myeloperoxidase, adenosine deaminase activity, and nitrite/nitrate concentrations) in the inflammation induced by carrageenan in the mouse model of pleurisy.

Groups	Doses (mg/kg)	MPO (mU/mL) (% of inhibition)	ADA (U/L) (% of inhibition)	NO_x_ (*μ*M) (% of inhibition)
Sal	0.9%^a^	26.37 ± 0.81	1.51 ± 0.04	12.63 ± 1.35
Cg	1%^a^	165.30 ± 7.61	6.22 ± 0.22	91.46 ± 3.83
CE	50^b^	105.50 ± 4.85 (36.03 ± 2.94%)^∗∗^	2.98 ± 0.35 (52.09 ± 5.65%)^∗∗^	35.73 ± 1.93 (60.93 ± 2.11%)^∗∗^
AqF	25^b^	98.47 ± 4.84 (40.32 ± 2.93%)^∗∗^	1.87 ± 0.33 (69.91 ± 6.87%)^∗∗^	37.05 ± 6.84 (59.49 ± 7.48%)^∗∗^
EtOAcF	10^b^	88.95 ± 5.29 (46.09 ± 3, 20%)^∗∗^	1.89 ± 0.32 (69.59 ± 6.73%)^∗∗^	34.72 ± 2.32 (62.04 ± 3.28%)^∗∗^
BuOHF	10^b^	106.0 ± 6.65 (35.75 ± 5.21%)^∗∗^	2.65 ± 0.27 (57.52 ± 4.39%)^∗∗^	42.04 ± 1.88 (54.03 ± 2.06%)^∗∗^
CUR	2.5^b^	102.70 ± 5.99 (37.74 ± 3.63%)^∗∗^	4.23 ± 0.19 (45.1 ± 8.9%)^∗∗^	32.11 ± 2.22 (65.67 ± 2.96%)^∗∗^
Pip	1^b^	100.1 ± 3.79 (39.35 ± 2.30%)^∗∗^	3.07 ± 0.35 (29.5 ± 11.7%)^∗∗^	31.14 ± 5.66 (61.76 ± 5.87%)^∗∗^
Dex	0.5^b^	104.60 ± 3.22 (36.59 ± 1, 95%)^∗∗^	2.48 ± 0.15 (60.23 ± 3.10%)^∗∗^	15.04 ± 1.38 (83.56 ± 1.50%)^∗∗^

Crude extract (CE: 50 mg/kg) of *Jungia sellowii* Less., aqueous fraction (AqF 25 mg/kg), butanol fraction (BuOHF 10 mg/kg), ethyl acetate fraction (EtOAcF: 10 mg/kg), curcuhidroquinone *O*-*β*-glucose (CUR: 2.5 mg/kg), and piptizol (Pip: 1 mg/kg) administered 0.5 h before pleurisy induction by carrageenan (1%). Sal: animals treated only with sterile saline solution (NaCl, 0.9%); Cg: animals treated only with carrageenan (1%); Dex: animals pretreated with dexamethasone (0.5 mg/kg); ^a^administered by intrapleural injection (i.pl.); ^b^administered by intraperitoneal route (i.p.). Each group represents the mean ± SEM of 5 animals compared to the positive control group (Cg); ANOVA/Newman-Keuls's test. ^∗^*p* < 0.05; ^∗∗^*p* < 0.01.

**Table 3:** Effects of the crude extract (CE) of *Jungia sellowii* Less., its derived fractions (aqueous (AqF), butanol (BuOHF), and ethyl acetate (EtOAcF)), and isolated compounds (curcuhidroquinone *O*-*β*-glucose (CUR) and piptizol (Pip)), upon cytokine concentrations, in the inflammation induced by carrageenan in the mouse model of pleurisy.

Groups	Doses (mg/kg)	TNF-*α* (pg/mL) (% of inhibition)	IFN-*γ* (pg/mL) (% of inhibition)	IL-6 (pg/mL) (% of inhibition)	IL-12 (pg/mL) (% of inhibition)
Sal	0.9%^a^	9.20 ± 3.25	25.0 ± 0.50	15.50 ± 2.96	13.0 ± 0.70
Cg	1%^a^	1252.00 ± 144.09	91.60 ± 8.40	1878.00 ± 37.84	363.70 ± 38.2
CE	50^b^	945.10 ± 89.48 (24.51 ± 7.15)^∗^	11.11 ± 2.60 (86.61 ± 2.87)^∗∗^	110.08 ± 3.89 (86.51 ± 5.03)^∗∗^	35.0 ± 2.5 (90.38 ± 0.85%)^∗∗^
AqF	10^b^	426.8 ± 60.3 (65.91 ± 4.82%)^∗∗^	9.0 ± 1.10 (90.21 ± 1.15)^∗∗^	209.10 ± 132.5 (82.90 ± 8.30)^∗∗^	31.50 ± 6.60 (91.33 ± 2.33%)^∗∗^
EtOAcF	10^b^	824.9 ± 141.4 (34.11 ± 14.58)^∗∗^	23.50 ± 1.20 (74.36 ± 1.64%)^∗∗^	110.3 ± 10.25 (94.13 ± 0.74)^∗∗^	90.60 ± 9.20 (75.10 ± 3.26%)^∗∗^
BuOHF	10^b^	179.2 ± 50.52 (85.68 ± 5.21)^∗∗^	11.5 ± 0.40 (87.40 ± 0.60)^∗∗^	72.75 ± 4.89 (96.13 ± 0.34)^∗∗^	236.00 ± 8.4 (35.11 ± 2.98%)^∗∗^
CUR	2.5^b^	465.30 ± 28.15 (62.83 ± 2.90%)^∗∗^	21.50 ± 0.40 (76.62 ± 8.92)^∗∗^	96.79 ± 23.29 (94.85 ± 1.60)^∗∗^	10.7 ± 0.70 (97.05 ± 3.2)^∗∗^
Pip	1.0^b^	978.2 ± 73.81 (21.81 ± 7.61)^∗^	12.0 ± 1.40 (86.87 ± 2.02)^∗∗^	119.50 ± 4.02 (93.63 ± 0.28)^∗∗^	96.80 ± 28.60 (73.38 ± 10.17%)^∗∗^
Dex	0.5^b^	18.86 ± 4.17 (98.49 ± 0.43)^∗∗^	27.5 ± 0.20 (85.45 ± 0.32)^∗∗^	42.77 ± 14.77 (97.72 ± 1.02)^∗∗^	33.90 ± 7.50 (90.69 ± 2.67%)^∗∗^

TNF-*α*: tumor necrosis factor alpha; IFN-*γ*: interferon gamma; IL-6: interleukin-6; IL-12: interleukin-12. Crude extract (CE: 50 mg/kg) of *Jungia sellowii* Less., aqueous fraction (AqF 25 mg/kg), butanol fraction (BuOHF: 10 mg/kg), ethyl acetate fraction (EtOAcF: 10 mg/kg), curcuhidroquinone *O*-*β*-glucose (CUR: 2.5 mg/kg), and piptizol (Pip: 1 mg/kg) administered 0.5 h before pleurisy induction by carrageenan (1%). Sal: animals treated only with sterile saline solution (NaCl, 0.9%); Cg: animals treated only with carrageenan (1%); Dex: animals pretreated with dexamethasone (0.5 mg/kg). ^a^Administered by intrapleural injection (i.pl.); ^b^administered by intraperitoneal route (i.p.). Each group represents the mean ± SEM of 5 animals compared to the positive control group (Cg); ANOVA/Newman-Keuls's test. ^∗^*p* < 0.05; ^∗∗^*p* < 0.01.

## Data Availability

The data used to support the findings of this study are included in the article.
